# Association between white matter structural damage and cognitive impairment in patients with cerebral small vessel disease based on TBSS technology

**DOI:** 10.3389/fneur.2025.1647129

**Published:** 2025-10-09

**Authors:** Qingqing Zheng, YaXi Luo, Yunchun Wang, Chengxi Yan, GuoWei Zhou, Min Ai

**Affiliations:** ^1^Department of Anesthesiology, Nanan District People’s Hospital of Chongqing, Chongqing, China; ^2^Department of Radiology, The First Affiliate Hospital of Chongqing Medical and Pharmaceutical College (The Sixth People’s Hospital of Chongqing), Chongqing, China; ^3^Department of Radiology, Renji Hospital, School of Medicine, Chongqing University (The Fifth People’s Hospital of Chongqing), Chongqing, China; ^4^Medical Imaging Department, Beijing Anzhen Nanchong Hospital of Capital Medical University & Nanchong Central Hospital, Sichuan, China; ^5^Department of Radiology, The Second Affiliated Hospital of Xi’an Jiaotong University, Shaanxi, China

**Keywords:** cerebral small vessel disease, cognitive impairment, diffusion tensor imaging, tract-based spatial statistics, multivariate logistic regression, risk prediction

## Abstract

**Objective:**

Cognitive impairment in patients with cerebral small vessel disease (CSVD) is closely associated with white matter injury. This study aims to evaluate whether diffusion tensor imaging (DTI) metrics can predict the risk of cognitive impairment in CSVD patients.

**Methods:**

We retrospectively analyzed data from 54 CSVD patients, classified into a cognitive impairment group (CI, *n* = 25) and a non-cognitive impairment group (NCI, *n* = 29). Using tract-based spatial statistics (TBSS), we computed fractional anisotropy (FA), mean diffusivity (MD), axial diffusivity (AD), and radial diffusivity (RD) across 48 major white matter tracts. Significant DTI metrics identified by univariate logistic regression were used to construct a multivariate logistic regression model. Model performance was evaluated via 5-fold cross-validation based on the area under the ROC curve (AUC), calibration curves, and decision curve analysis.

**Results:**

Several DTI metrics showed significant correlations with cognitive impairment, including FA (fornix, left corticospinal tract, bilateral medial lemniscus/inferior cerebellar peduncle, left cerebral peduncle, right cingulum hippocampus), MD (right superior cerebellar peduncle, left cerebral peduncle), and RD (bilateral medial lemniscus, right inferior/superior cerebellar peduncle, left cerebral peduncle, right external capsule, cingulum hippocampus). The multivariate model constructed based on these metrics demonstrated the best predictive performance, with a mean training AUC of 0.940 and testing AUC of 0.809. The calibration curves showed good agreement between predicted and observed outcomes, and decision curve analysis confirmed the clinical utility of the model.

**Conclusion:**

The multivariate logistic regression model incorporating DTI metrics can effectively identify cognitive impairment in CSVD patients. This study establishes a link between damage in specific white matter tracts and cognitive dysfunction, providing a practical tool for assessing the risk of cognitive impairment in clinical settings.

## Introduction

Cerebral small vessel disease (CSVD) is a group of disorders characterized by pathological changes in small arteries, arterioles, capillaries, and venules within the brain, manifesting with diverse clinical symptoms including cognitive impairment, gait abnormalities, and mood disorders (1). Cognitive impairment is a common symptom among CSVD patients, with some progressing to dementia while others maintain relatively normal cognitive function (2). This heterogeneity suggests the existence of distinct pathophysiological mechanisms within the CSVD patient population. Consequently, identifying cognitive impairment in CSVD patients is crucial not only for understanding the heterogeneity of disease progression but also for providing a basis for personalized treatment and early intervention.

Clinically, the diagnosis and classification of CSVD with cognitive impairment have largely relied on conventional neuroimaging markers—such as white matter hyperintensities (WMH), lacunes, cerebral microbleeds (CMBs), and enlarged perivascular spaces (EPVS)—combined with cognitive assessment scales. While these markers provide valuable diagnostic information, they exhibit limitations in sensitivity and specificity, particularly in early stages of cognitive decline. The assessment often depends on subjective interpretation and may be influenced by patient compliance and rater experience, potentially leading to underdiagnosis or delayed intervention (3).

In recent years, advanced neuroimaging techniques have offered new insights into the microstructural changes underlying CSVD. Among these, diffusion tensor imaging (DTI) has emerged as a powerful tool for probing the integrity of white matter tracts, which are frequently compromised in CSVD (4). Unlike conventional MRI markers, which primarily capture macroscopic lesions, DTI-derived metrics—such as fractional anisotropy (FA), mean diffusivity (MD), axial diffusivity (AD), and radial diffusivity (RD)—provide quantitative measures of microstructural integrity, axonal density, and myelin organization. There is growing evidence that DTI parameters may serve as earlier and more sensitive indicators of cognitive decline than volumetric or lesion-based measures (5, 6), and the severity of white matter damage is a primary risk factor for cognitive impairment in CSVD (7). Therefore, given the limitations of clinical assessment and the established role of white matter damage, advanced neuroimaging techniques like DTI offer a promising avenue for objective assessment. Therefore, distinguishing CSVD patients with cognitive impairment from those without using conventional MRI presents significant challenges, highlighting the urgent need for an objective and accurate method.

Tract-based spatial statistics (TBSS), a voxel-wise analytical method for DTI data, enhances the detection of white matter alterations by aligning individual tracts to a common skeleton, thereby reducing misregistration and partial volume effects (8). This technique allows for a more precise evaluation of microstructural changes across the entire white matter architecture (9). Importantly, CSVD patients frequently exhibit damage to white matter tracts, which is considered a crucial pathological basis for cognitive impairment and serves as a sensitive MRI marker for monitoring disease progression and evaluating therapeutic interventions in CSVD (10). White matter tracts are critical pathways for information transfer between different brain regions, and their integrity is essential for maintaining normal cognitive function (11). Studies show that the extent of white matter tract damage in CSVD patients is closely correlated with cognitive decline. Therefore, investigating the patterns of white matter tract damage in CSVD can help elucidate the neural mechanisms underlying cognitive impairment (12, 13).

Building on this rationale, in this study, we employed TBSS to analyze DTI data from CSVD patients with and without cognitive impairment, extracting FA, AD, MD, and RD values. Univariate logistic regression analysis was then used to identify regions of white matter tract damage that differed between the two groups. Subsequently, a multivariate logistic regression model was constructed using the FA, AD, MD, and RD data from these differential regions to distinguish the two patient groups. Furthermore, we utilized nomograms to visualize the relevant risk factors and quantify the predictive value of each indicator for cognitive impairment in patients.

This research not only contributes to a deeper understanding of the pathophysiological mechanisms of CSVD but also provides a scientific basis for the early identification of high-risk patients and the formulation of personalized intervention strategies, holding significant clinical relevance. By constructing multivariate logistic regression models, we aim to offer new approaches for the precise diagnosis and treatment of CSVD patients.

## Materials and methods

This retrospective study was approved by the Ethics Committee of the Second Affiliated Hospital of Chongqing Medical University, and written informed consent was obtained from all participants. A total of 54 patients diagnosed with cerebral small vessel disease (CSVD) were enrolled. Diagnoses were confirmed by neurologists with specialized training.

### Inclusion criteria

(1) MRI evidence of CSVD in one or both brain hemispheres; (2) Assessment of global cognitive function using cognitive assessment scales, with objective evidence of impairment in at least one cognitive domain, or no objective evidence of global cognitive impairment; (3) Corresponding neuroimaging diagnoses of recent small subcortical infarcts (RSSIs), cerebral microbleeds (CMBs), enlarged perivascular spaces (EPVS, grade 2–4), cortical superficial siderosis (cSS), and white matter hyperintensities (WMH, Fazekas score ≥2); (4) Undergone cranial MRI and DTI scanning.

### Exclusion criteria

(1) Acute intracranial large vessel diseases, such as ischemic stroke or cerebral hemorrhage; (2) Metabolic encephalopathy, hypoxic-ischemic encephalopathy, or other non-vascular white matter lesions; (3) Other concurrent intracranial pathologies, such as tumors, dementia, head trauma, or other diseases; (4) Incomplete clinical data; (5) Incomplete imaging data; (6) Severe imaging artifacts.

**Figure 1:**
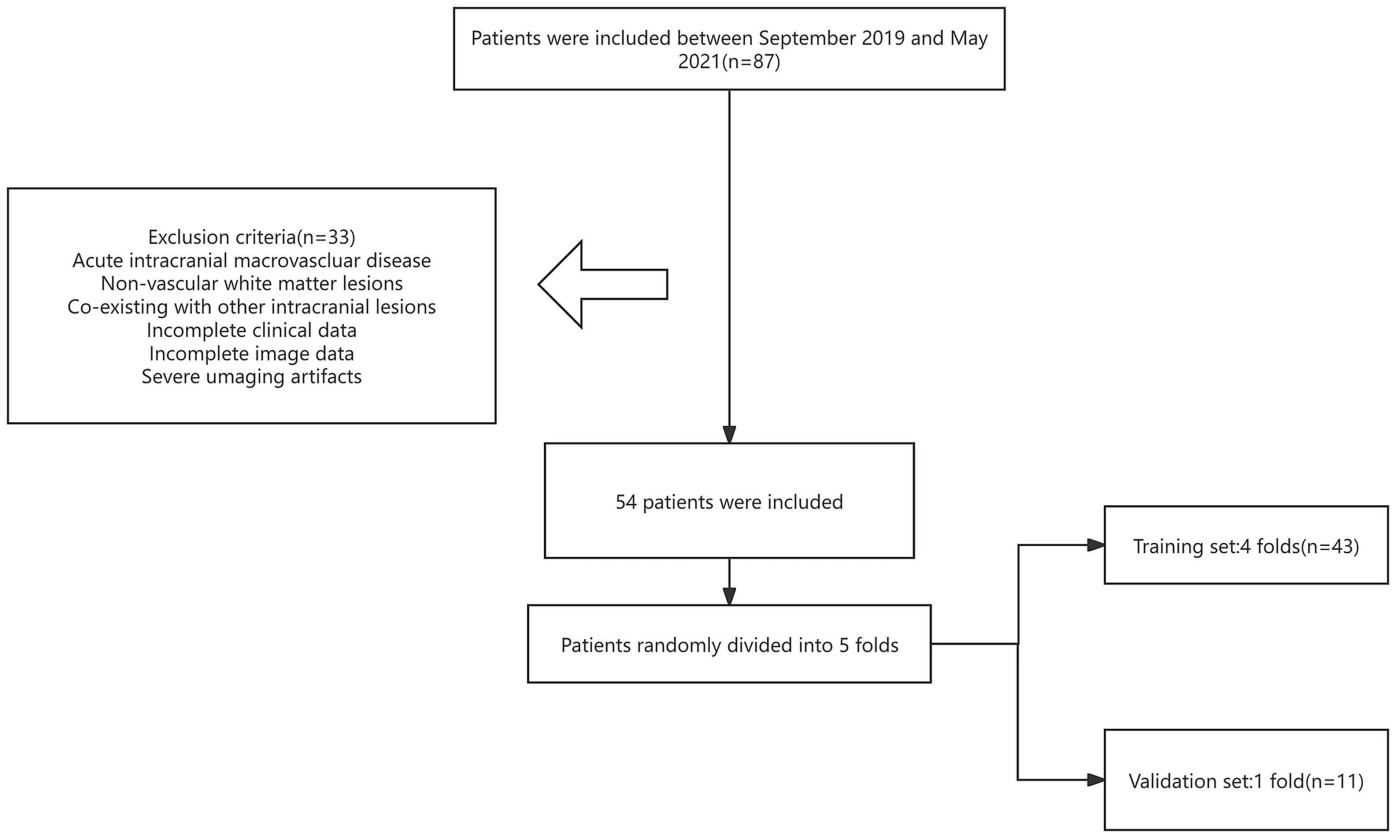
Flow chart of subject recruitment.

All patients underwent clinical evaluation prior to MRI scanning. Demographic information (age and sex) was recorded, and cognitive assessment was performed using the Chinese version of the Mini-Mental State Examination (MMSE) and the Montreal Cognitive Assessment (MoCA) (14). Both MMSE and MoCA were administered face-to-face by experienced neurologists strictly adhering to guidelines and protocols. Participants’ MoCA and MMSE scores were used for patient group classification. The flow chart of subject recruitment was shown in Figure 1.

### MRI examination

#### Scanning parameters

MRI data for all participants were acquired using a 3.0 T MRI scanner (Achieva 3.0 T, Philips, Netherlands) equipped with a 32-channel head coil. The scanning protocol included T1-weighted imaging (T1WI) and DTI. T1WI parameters were: repetition time (TR) = 7.9 ms, echo time (TE) = 39 ms, field of view (FOV) = 256 × 256 mm^2^, matrix = 256 × 256, slice thickness = 1 mm, interslice gap = 0 mm. DTI parameters were: TR/TE = 6,000/70 ms; FOV = 256 × 256 mm^2^; voxel size = 2.50 × 2.50 × 2.50 mm^3^. Each DTI dataset included 32 images acquired with non-collinear diffusion gradients at *b* = 1,000 s/mm^2^ and one baseline image at *b* = 0 s/mm^2^.

### Processing and data analysis

#### Data preprocessing

DICOM format images were converted to NIFTI format using dcm2niix software.

Using FSL (FMRIB Software Library v6.0; https://fsl.fmrib.ox.ac.uk/fsl/fslwiki/FSL), the fsl roi command was used to extract the b0 image. The bet command was used for brain extraction, generating a brain mask. The eddy_correct command was applied to correct the DTI data (NIFTI format) for eddy current distortions and head motion. Following these preprocessing steps, the dtifit tool was used to generate voxel-wise diffusion parameter maps: fractional anisotropy (FA), mean diffusivity (MD), axial diffusivity (AD), and radial diffusivity (RD).

#### TBSS analysis

The FA maps were non-linearly registered to the FMRIB58_FA_1mm standard template in MNI space using a two-step procedure. A mean FA skeleton was created, thresholded at FA >0.2. This mean skeleton was then projected onto each subject’s FA map (in standard space) to create individual skeletonized FA maps. To ensure consistent spatial analysis across all diffusion metrics, the transformation parameters derived from FA registration were applied to the MD, AD, and RD maps to generate their corresponding skeletonized maps (15).

### Statistical analysis

#### Data preprocessing

All statistical analyses were performed using R software (version 3.6.3) and the randomise tool within FSL. Normally distributed continuous variables are expressed as mean ± standard deviation (SD). Group comparisons for continuous variables were assessed using the *t*-test. Multiple comparison correction is performed using TFCE (threshold-free cluster enhancement correction). Categorical variables were compared using the Chi-square test or Fisher’s exact test.

Voxelwise statistical analysis of the skeletonised FA, MD, AD, and RD data was carried out using FSL’s randomise tool (version 6.0), which employs a non-parametric permutation testing framework to avoid assumptions of normality. Group differences (CI vs. NCI) for each diffusion metric (FA, MD, AD, RD) were tested using a general linear model (GLM) with 5,000 permutations.

To address the multiple comparisons problem inherent in voxelwise analysis across the entire white matter skeleton, we applied threshold-free cluster enhancement (TFCE). TFCE is a method that enhances the signal-to-noise ratio of cluster-like structures without relying on an arbitrary initial cluster-forming threshold. It transforms the raw statistic image by integrating cluster support at every point, providing a refined output image where the value at each voxel represents a combination of the height and spatial extent of the signal. This TFCE-transformed image is then used for inference, making the method more sensitive and robust compared to traditional cluster-based thresholding.

The significance of the TFCE-corrected results was assessed by comparing the observed test statistics to the null distribution generated from the permuted data. Results were considered statistically significant at a family-wise error (FWE) corrected *p*-value < 0.01.

### Modeling

To develop and validate predictive models while accounting for the limited sample size and enhancing the robustness of the evaluation, we employed a 5-fold cross-validation strategy. The entire cohort of CSVD patients was randomly partitioned into 5 subsets of approximately equal size. In an iterative process, four subsets were used as the training set to develop the model, and the remaining subset was used as the testing set for validation. This process was repeated 5 times, ensuring that each subset served as an independent test set exactly once.

Univariate logistic regression analyses were performed on the diffusion tensor data from the entire cohort to identify regions and parameters significantly associated with cognitive impairment (*p* < 0.01). Variables with statistical significance in the univariate analysis, along with clinical covariates such as age, educational level, and white matter hyperintensity, were incorporated into a multivariable logistic regression model for construction. Variables that showed significant associations in the univariate analysis were subsequently included in the multivariate logistic regression model. The performance of the established models was evaluated by averaging the results across all 5 folds. The evaluation metrics included the area under the receiver operating characteristic curve (AUC), calibration curves, and decision curve analysis (DCA). The final model was visualized using a nomogram.

## Results

### Demographic and clinical characteristics of patient groups

This retrospective study examined 54 CSVD patients. Among them, 25 had cognitive impairment (CI) and 29 did not (NCI). Comparison of baseline characteristics revealed no statistically significant differences between the two groups in terms of sex, smoking status, prevalence of hypertension or diabetes, or education level (Supplementary Table 1). However, a significant difference was observed in patient age. Demographic and clinical characteristics of training and test sets: There were no statistically significant differences in variables between the training and test sets.

### Variable selection based on univariate logistic regression and model performance

**Figure 2:**
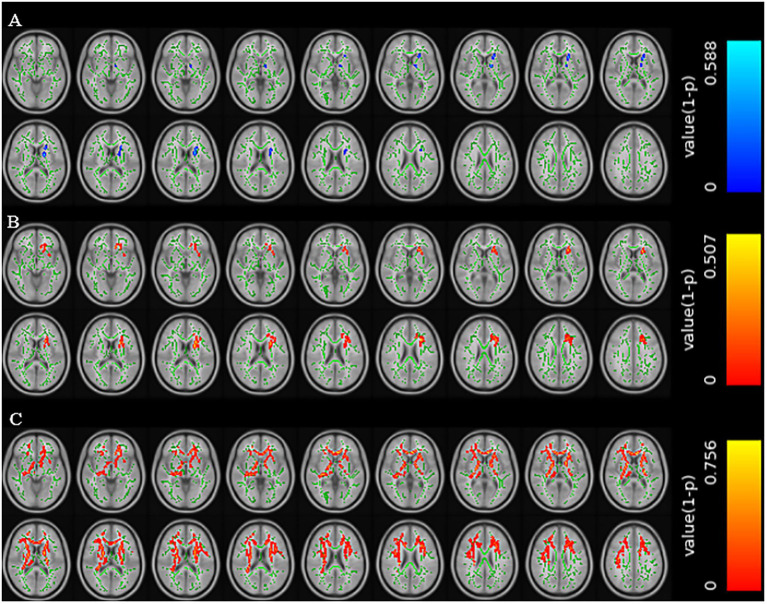
Brain regions with reduced FA values in the CSVD group with cognitive impairment **(A)**. Brain regions with increased MD values in the CSVD group with cognitive impairment **(B)**. Brain regions with increased RD values in the CSVD group with cognitive impairment **(C)** (TFCE correction, *p* < 0.01).

Compared with patients without cognitive impairment, patients with cognitive impairment showed decreased FA values in the left anterior limb of the internal capsule, left posterior limb of the internal capsule, left anterior radiation, and left superior radiation (TFCE corrected, *p* < 0.01); increased MD values in the genu of the corpus callosum, body of the corpus callosum, left anterior limb of the internal capsule, left anterior radiation, left superior radiation, left external capsule, and left superior fronto-occipital fasciculus (TFCE corrected, *p* < 0.01); and increased RD values in multiple regions including the genu and body of the corpus callosum, bilateral anterior and posterior limbs of the internal capsule, bilateral superior and anterior radiation, and several other tracts (TFCE corrected, *p* < 0.01) (Figure 2).

To formally identify significant predictors, univariate logistic regression analysis was performed between the extracted DTI parameters (FA, RD, AD, MD) and cognitive impairment. Significant associations (*p* < 0.01) were found between CI and FA parameters in several regions, including the corpus callosum (body), left corticospinal tract, bilateral medial lemnisci, and bilateral inferior cerebellar peduncles. Significant associations were also found for MD and RD parameters in specific regions, while no significant associations were found for AD parameters.

**Figure 3:**
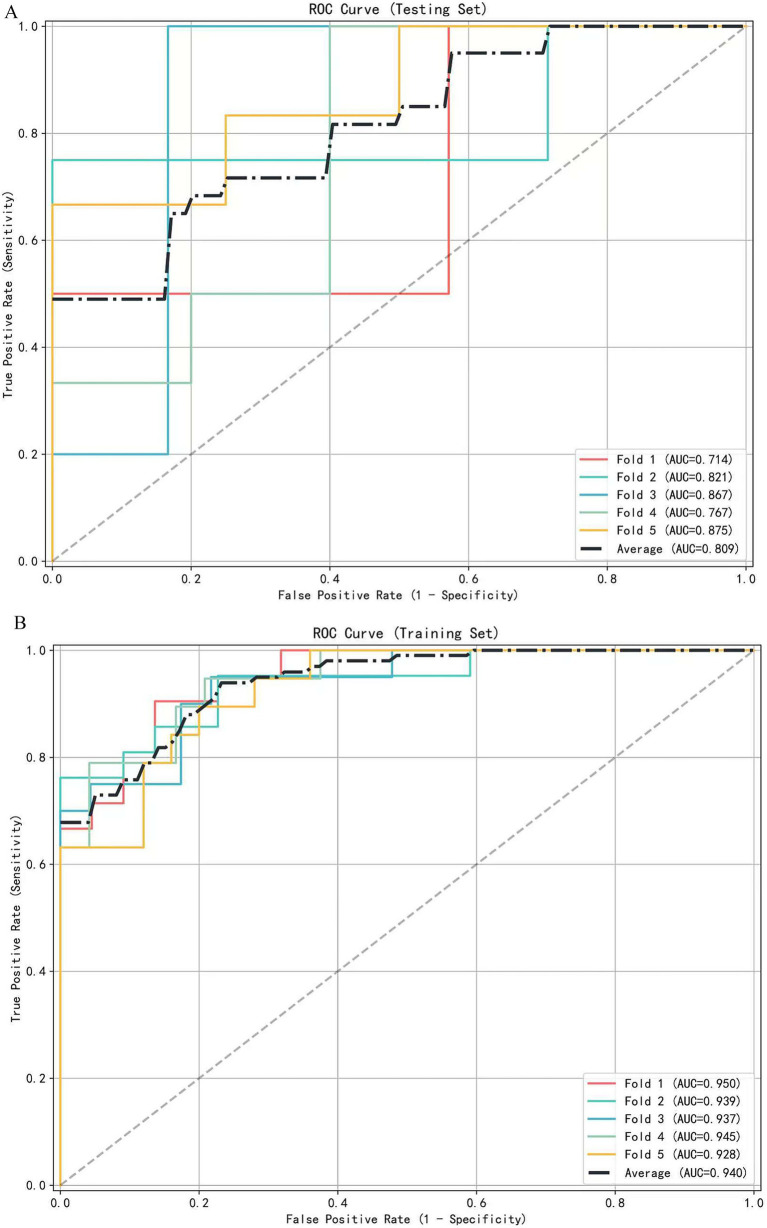
The receiver operating characteristic (ROC) curve of the diagnostic model is based on 5-fold cross-validation. **(A)** ROC curves for the testing set (mean AUC = 0.809). **(B)** ROC curves for the training set (mean AUC = 0.940).

**Figure 4:**
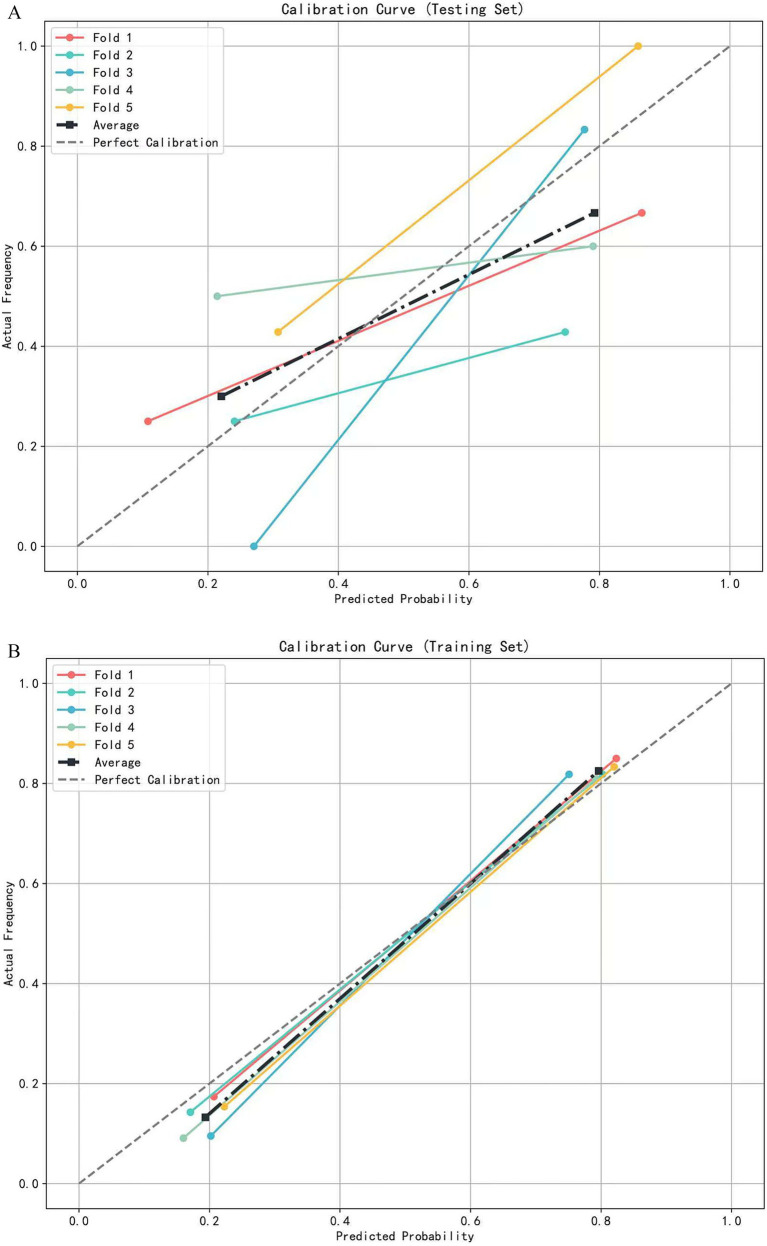
The calibration curve of the logistic regression model based on 5-fold cross-validation. **(A)** Calibration curves for the testing set. **(B)** Calibration curves for the training set.

**Figure 5:**
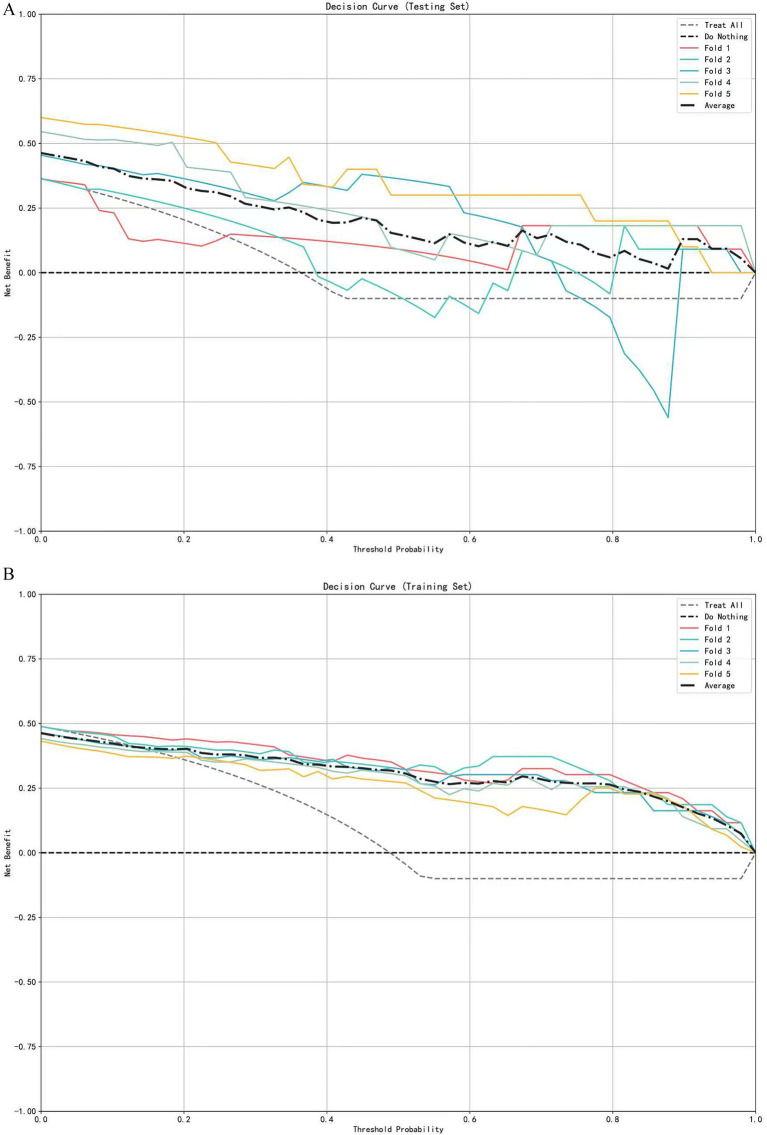
Decision curve analysis of the logistic regression model based on 5-fold cross-validation. **(A)** Decision curves for the testing set. **(B)** Decision curves for the training set.

**Figure 6:**
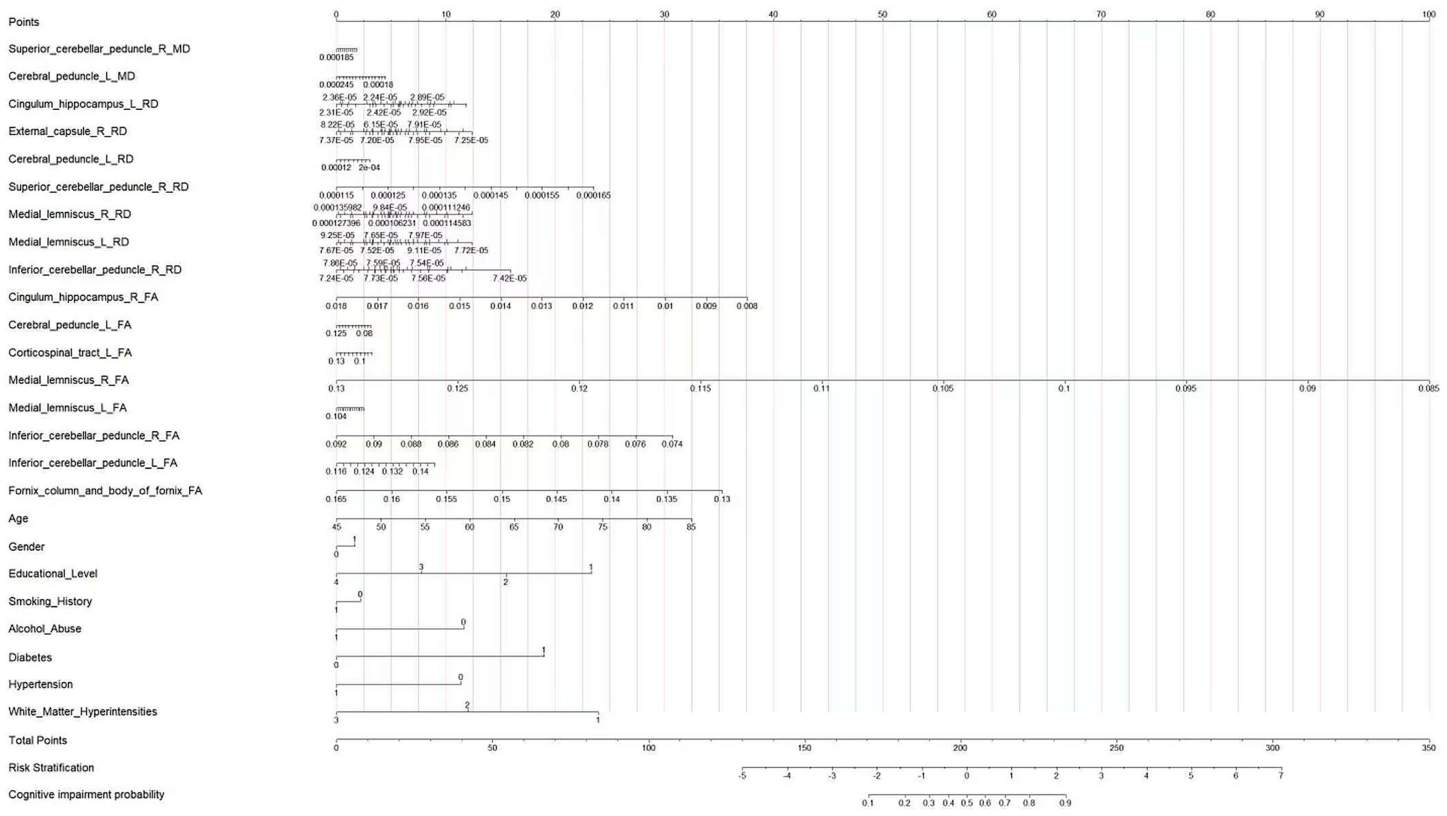
Nomogram for predicting the probability of cognitive impairment in patients with CSVD.

A multivariate logistic regression model was constructed using the above-identified significant fractional anisotropy (FA), radial diffusivity (RD), and mean diffusivity (MD) variables, combined with clinical variables (such as age). A 5-fold cross-validation was employed to evaluate the model. The average area under the curve (AUC) values from cross-validation for the training set and test set were 0.940 and 0.809, respectively (Figure 3). The calibration curves of both the training set and test set demonstrated good consistency between the actual probabilities and predicted probabilities of the samples (Figure 4). The Hosmer–Lemeshow test indicated good model fit (*p* > 0.05 for both training and test sets). Variance inflation factors (VIFs) for all predictor variables were below 5, indicating no significant multicollinearity. Decision curve analysis indicated that the model yielded high clinical benefits for both the training set and test set within a wide range of threshold probabilities (Figure 5). The nomogram incorporated the following risk predictors: FA parameters of the corpus callosum (body part), left corticospinal tract, bilateral medial lemniscus, bilateral inferior cerebellar peduncles, left cerebral peduncle, and right cingulum (hippocampal part); MD parameters of the right superior cerebellar peduncle and left cerebral peduncle; RD parameters of the bilateral medial lemniscus, right inferior cerebellar peduncle, right superior cerebellar peduncle, left cerebral peduncle, right external capsule, and left cingulum (hippocampal part); as well as the patients’ clinical data (Figure 6).

### Interpretation of the nomogram

This nomogram integrates diffusion tensor imaging (DTI) parameters of cerebral white matter fiber tracts, demographic characteristics, educational level, and health-related factors to quantitatively calculate the probability of an individual developing cognitive impairment. First, obtain the specific values of each predictive variable of the patient. Then, in the row corresponding to each variable, find the corresponding score on the “Points” axis (0–100 scale) according to its value. Next, sum up the scores corresponding to all predictive variables to get the “Total Points” (total score, 0–350 scale). Finally, find the corresponding position on the “Cognitive impairment probability” axis according to the total score, and the probability of the patient developing cognitive impairment can be obtained.

Clinicians can use this nomogram to integrate various information of patients and quantitatively assess their risk of developing cognitive impairment. For patients at high risk, intervention measures can be formulated in advance, such as strengthening cognitive function monitoring and conducting cognitive training; for patients at low risk, an appropriate follow-up plan can be formulated. At the same time, this diagram also provides a visualized tool that integrates multiple factors for studying the pathogenesis of cognitive impairment, which is helpful for in-depth understanding of the impact of different factors on the occurrence of cognitive impairment.

## Discussion

With global population aging, the prevalence of CSVD and its associated cognitive decline is projected to rise substantially, imposing a considerable burden on healthcare systems. Furthermore, studies have demonstrated that early identification of individuals at risk of developing cognitive impairment and timely clinical intervention can reduce the incidence of dementia in CSVD patients. This underscores the urgent need to develop early diagnostic biomarkers and tools for assessing cognitive impairment risk in the context of CSVD-related cognitive decline (16).

Previous research has shown that loss of microstructural integrity in specific white matter regions correlates with specific cognitive dysfunctions in CSVD patients (17), highlighting the crucial role of white matter in cognitive decline (18, 19). Increasing evidence suggests that DTI parameters serve as earlier markers of cognitive decline compared to volumetric measures. Tract-based spatial statistics (TBSS) applied to DTI analysis mitigates the influence of cerebrospinal fluid (CSF) partial volume effects by focusing on the core skeleton of white matter tracts (20). In line with this approach, in this study, we investigated CSVD patients with and without cognitive impairment. Utilizing TBSS-extracted parameters, we ultimately constructed classification models based on DTI parameters. The results indicate that DTI parameters can serve as a tool for distinguishing CSVD patients with cognitive impairment from those without. A key finding was that FA parameters demonstrated a broader range of associations as predictors of cognitive impairment risk compared to other DTI parameters. Specifically, when we explored the contribution of different DTI parameters individually for distinguishing the two patient groups. We found that for FA, we identified differences in 9 regions between the two groups, more than found for other parameters. This suggests FA may be more sensitive than other metrics for monitoring white matter disruption in CSVD and more strongly correlated with cognitive scales. This observation contrasts with some prior studies that have reported that AD might be more sensitive for detecting cognitive impairment (21), while others align with studies suggesting MD is more sensitive in mild cognitive impairment (22, 23).

The primary early pathology in cerebral small vessel disease (CSVD) may not be purely axonal injury: numerous studies have shown that one of the earliest changes in CSVD is blood–brain barrier disruption and glial cell pathology, which is followed by demyelination. Radial diffusivity (RD) is more sensitive to myelin changes. As a comprehensive indicator, fractional anisotropy (FA) captures changes in both axial diffusivity (AD, related to axons) and RD (related to myelin) simultaneously. In our cohort, if demyelination is the more dominant pathological process, the signals from FA (and RD) may mask the contribution of AD, resulting in AD itself being non-significant in the model. This is consistent with several studies that emphasize the key role of myelin injury in cognitive decline associated with CSVD.

Importantly, we found that for FA, patients with CI showed damage in the cingulum (hippocampal part) and fornix regions compared to the NCI group. RD also indicated damage in the cingulum (hippocampal part) in the CI group. This finding is particularly noteworthy because previous research on Alzheimer’s disease (AD) has reported that the cingulum (hippocampal part) and fornix are often vulnerable in the preclinical AD stage and are highly associated with memory function (24). While processing speed and executive function are typically considered the most vulnerable cognitive domains in cerebrovascular disease, and unlike AD, CSVD progressively affects all major cognitive domains. Therefore, our results suggest that damage to the cingulum (hippocampal part) and fornix white matter tracts is also highly relevant to cognition in CSVD patients, potentially explaining broader cognitive deficits beyond pure executive dysfunction.

Our study also revealed white matter tract damage in the external capsule region between the groups. The significance of this region lies in the fact that the white matter fibers of the external capsule form a significant part of the lateral pathway of the cholinergic system, projecting to the dorsal frontoparietal neocortex, temporal cortex, and parahippocampal gyrus (25). This finding aligns with growing interest in cholinergic dysregulation in CSVD. Indeed, one tractography study found significantly reduced FA within cholinergic pathways (including the external capsule, cingulum, and claustrum) in patients with vascular cognitive impairment no dementia (26). Damage to these pathways can adequately explain executive dysfunction and partially account for memory and global cognitive impairment. Another tractography study identified the external capsule as the lateral cholinergic bundle and found diffusion metrics in the external capsule and the overlying superior longitudinal fasciculus correlated with executive dysfunction (27). Thus, our findings support the hypothesis that the lateral cholinergic bundle is significantly associated with executive dysfunction in the early stages of cognitive decline in CSVD, compared to CSVD patients without cognitive impairment.

Interestingly, we identified damage in the cerebral peduncle and inferior cerebellar peduncle regions. The relevance of these findings stems from the role of the white matter tracts in these regions which participate in the dentato-rubro-thalamic and dentato-thalamic pathways, the primary connections between the cerebellum and cerebral cortex. These pathways link the “cognitive cerebellum” (including lobules VI, VII, VIIB, and Crus I), involved in language, verbal memory, spatial tasks, and executive function, with the associative cerebral cortex (28). This observation is consistent with increasing research highlighting the cerebellum’s role in cognitive regulation, and suggests that microstructural changes in cerebello-cerebral pathways may underlie the pathophysiological cerebellar alterations related to cognitive deficits revealed by resting-state fMRI in CSVD patients (29). Our observation of microstructural damage in cerebello-cerebral pathways (cerebral peduncle, inferior cerebellar peduncle) finds support in the work of Mascalchi et al. (30), who also reported significant correlations between MoCA scores and DTI alterations (reduced FA, increased MD/RD) in the decussation of the superior cerebellar peduncles in the midbrain (dentate-rubro-thalamic tracts) in their vascular MCI cohort. This convergence of evidence across independent studies strengthens the notion that disruption of cerebellar-cerebral connectivity is a clinically relevant component of cognitive impairment in cerebral small vessel disease, potentially contributing to deficits in executive function and processing speed (31).

Overall, our results highlight several key aspects: findings regarding damage to the cingulum (hippocampal part) and fornix, alongside potential cholinergic dysregulation, contribute to understanding cognitive decline in CSVD. Moreover, the practical application of this work is demonstrated by the logistic regression models we constructed, which provide a potential approach for clinically distinguishing CSVD patients with cognitive impairment, indicate the risk of cognitive impairment in CSVD patients, and offer some basis for clinicians’ personalized medical decisions.

Despite providing valuable insights, several limitations must be acknowledged. First, this is a cross-sectional study; longitudinal research is needed to elucidate the dynamic relationship between DTI parameters and cognitive impairment progression over time in CSVD patients. Secondly, the relatively small sample size and the failure to classify patients into cerebral small vessel disease (CSVD) subtypes may limit the generalizability of the study results. Therefore, larger-scale cohort studies are needed to validate and expand the study conclusions, and an external validation set is also an issue we consider. Finally, as comprehensive cognitive screening tools, the Mini-Mental State Examination (MMSE) and Montreal Cognitive Assessment (MoCA) lack sufficient depth in evaluating specific cognitive domains such as executive function. Supplementing with specific cognitive scales may more comprehensively reveal the characteristics of changes in CSVD-related cognitive impairment. Furthermore, the findings of this study should be regarded as a promising predictive tool, as the biological interpretation of diffusion-related imaging metrics in specific cognitive domains remains immature. Future research incorporating multimodal data (e.g., PET, histology) is still needed to further elucidate these relationships.

## Conclusion

In conclusion, our study demonstrates that classification models based on DTI parameters can distinguish CSVD patients with cognitive impairment from those without. Among these, the multivariate logistic regression model based on fractional anisotropy (FA) values performed best, effectively identifying CSVD patients with cognitive impairment. This study is the first to identify specific white matter tract damage patterns associated with cognitive impairment in CSVD patients and provides a tool for assessing cognitive impairment risk, holding significant clinical application value.

## Data Availability

The raw data supporting the conclusions of this article will be made available by the authors, without undue reservation.
