# Supplementary material for: Association between white matter structural damage and cognitive impairment in patients with cerebral small vessel disease based on TBSS technology
Source: Front Neurol. 2025 Oct 9;16:1647129. doi: 10.3389/fneur.2025.1647129 (PMC12545093; doi:10.3389/fneur.2025.1647129)
Supplement: Supplementary file 1 [file Table_1.docx]

Table 1 The demographic data of CSVD with or without cognitive impairment groups

| Variable | CSVD with cognitive impairment | CSVD without cognitive impairment | P-value |
| --- | --- | --- | --- |
| Sample size | 29 | 25 | / |
| Gender | / | / | / |
| Female | 19 | 19 | 0.2 |
| Male | 10 | 6 |  |
| Age | 65.7±9.4 | 71.8±7.5 | 0.012* |
| Education | / | / | / |
| llliteracy | 0 | 2 | 0.209 |
| primary | 4 | 6 |  |
| middle | 18 | 14 |  |
| university | 7 | 3 |  |
| Smoking | / | / | / |
| No | 25 | 22 | 0.845 |
| Yes | 4 | 3 |  |
| Alcohol abuse | / | / | / |
| No | 25 | 22 | 0.845 |
| Yes | 4 | 3 |  |
| Diabetes | / | / | / |
| No | 25 | 17 | 0.109 |
| Yes | 4 | 8 |  |
| Hypertension | / | / | / |
| No | 6 | 6 | 0.77 |
| Yes | 23 | 19 |  |

**Note:** The P-values for continuous variables were calculated using the two-sample t-test, and the P-values for categorical variables were calculated using the chi-square test or Fisher's exact test. * indicates that the P-value is less than 0.05.
